# Limb girdle muscular dystrophy: a case report initially presenting to an outpatient musculoskeletal physiotherapy clinic with spinal pain and functional weakness

**DOI:** 10.1186/s40945-019-0066-3

**Published:** 2019-11-14

**Authors:** Simon O’Shea, Thomas M. Jenkins

**Affiliations:** 1 PhysioWorks, Sheffield Teaching Hospitals NHS Foundation Trust, Bochum Parkway, Sheffield, S8 8JR England; 2 Department of Neurology, Sheffield Teaching Hospitals NHS Foundation Trust, Sheffield, England

**Keywords:** Limb girdle muscular dystrophy, Back pain, Weakness

## Abstract

**Background:**

The term limb girdle muscular dystrophy (LGMD) describes a group of genetic muscular disorders that require specialist input from neurologically trained clinicians. The plethora of potential symptoms of this heterogenous group can result in patients presenting initially to musculoskeletal (MSK) physiotherapists.

**Case presentation:**

The following case report highlights the presentation of a 21 year old female attending with 2 years of spinal pain and an unusual pattern of weakness, namely when rising from a sitting position the hips were abducted and then internally rotated. Formal testing in clinic revealed no isolated weakness initially despite the odd functional movements. There were no neural limb pains and no upper or lower motor neuron concerns on testing. There were no other health concerns. Some gains were reported with recent physiotherapy strengthening exercises and these were persisted with but proved ineffective overall. The Biopsychosocial model was used judiciously to explore alternative pathologies and led to appropriate investigations, onward referral, diagnosis and appropriate management of LGMD. Extensive atrophy of the spinal muscles was evident on imaging which was not particularly identified within the physiotherapy testing process in the earlier stages. Creatine kinase levels were also significantly raised.

**Conclusions:**

Being mindful of this novel presentation in musculoskeletal clinics may well aid future, similar cases to be identified. The case highlights the importance of looking at the functional impact as opposed to traditional testing methods especially in the early stages of such conditions.

## Background

The following case report highlights a serious pathology in the form of limb girdle muscular dystrophy (LGMD). The patient presented initially to a musculoskeletal (MSK) outpatient clinic with back pain but with atypical associated features. Dissemination of such a case may benefit clinicians. The use of diagnostics is discussed, such as magnetic resonance imaging (MRI) and creatine kinase blood testing. There was a potential risk of mislabelling the patient if such rare neurological conditions were not considered. This case report highlights the need for careful consideration of pathological origins outside of the clinicians niche. The lack of benefit from physiotherapy treatment and re-evaluation of the case enabled such pathology to be investigated and specialist care initiated in a timely manner. The following report highlights key features of the presentation and the challenges of initial clinical examination and investigations. It also aims to raise the awareness of LGMD to MSK clinicians.

## Case presentation

### Present history

**Fig. 1:**
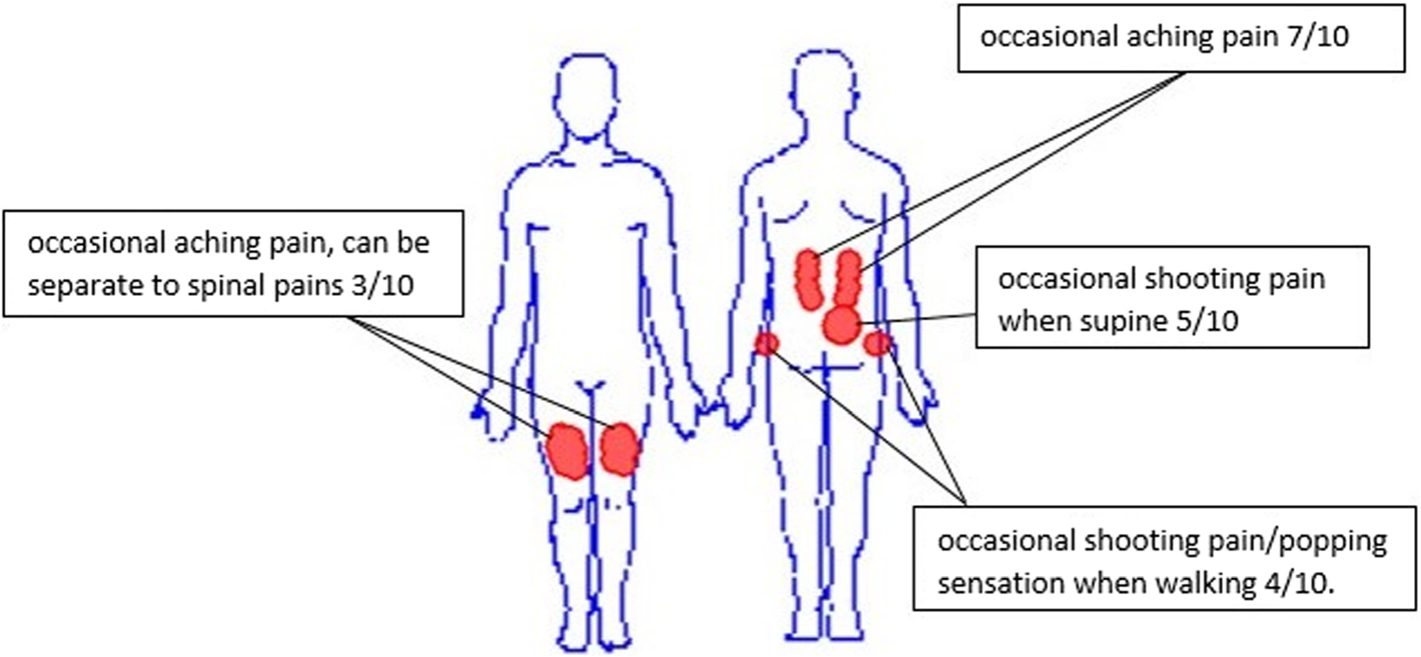
Body chart of case report

A 21 year-old female undergraduate student presented with a 2 year history of spinal, pelvic and distal thigh pains with an insidious onset. There were no reports of neurogenic-type pain or sensory changes. See Fig. 1 for the body chart.

The patient reported a slight weakness negotiating a flight of stairs but she was unsure if she felt weakness in her lower extremities or her trunk. Aggravating factors included walking up more than down stairs, sit to stand transfers and stepping up curbs. In sitting, no symptoms were evident.

There was no history of weight change, night sweats or recent infections/systemic illness. Early morning stiffness was described as minimal but lasted 40 min. There was no family history of any significance. The patient recalled always being last at running races as a child but no missed milestones or paediatric input was highlighted. Medication included naproxen and paracetamol as required, taken a few times per week with mild relief. No other medical, drug or mental health history was noted. Attendance at University had not been interrupted. No change to social activities or family relationships were reported due to the symptoms.

Past treatment included recent private physiotherapy and the patient’s interpretation was that “core exercises” had been initiated. These had been performed twice a day for 2 weeks. Subjectively, pain severity and the sense of strength when negotiating stairs had improved by 10% since starting the exercises. Care was transferred to the authors NHS service as is typical in the UK to enable free provision of assessment and treatment.

### Questionnaire responses

A 9-question tool called The Subgroups for Targeted Treatment (STarT) back screening tool questions patients on matters such as catastrophising, fear, anxiety and depression [1]. The tool has been advised to identify back pain patients who may have modifiable aspects to their presentation that may benefit from cognitive-behavioural approaches [1].

The initial STarT back score was 7 with a subscore of 4 (see Additional file 1) indicating a high risk factor for spinal pain-related disability [1]. The score indicates significant psychosocial risk factors for a poor prognosis [1] and stratification to physiotherapy services to deal with psychosocial elements would be advised [1–3]. The EuroQol 5D (EQ. 5D) questionnaire gives health related quality of life and psychometric analysis that has demonstrable construct validity and reliability [4]. The patients EQ. 5D responses demonstrated severe pain, functional impact and anxiety/depression scores (see Additional file 2).

### Examination

Subtle spinal rotation and thoracic scoliosis concave to the right was noted on standing. The gait pattern was entirely normal including rope walking tasks. The sit to stand movement pattern was abnormal. A wide base of support was achieved by abducting the hips. The lower limbs were internally rotated and the upper limbs were used to assist rising from the chair.

Dermatomes, reflexes and tone were deemed normal. There were no Babinski or Hoffmann’s reflexes. There was no clonus at the feet. The Romberg’s test was also negative and there was no dysdiadochokinesis. Myotomal examination revealed no focal weakness when examined in isolation, but weakness was apparent during sit to stand and bridging tasks. Single leg stance demonstrated a good ability to maintain pelvic and trunk posture with no Trendelenburg. There was no visible muscle atrophy but a mildly raised BMI may have limited the ability to detect a loss of muscle bulk.

Lumbar spine movements into extension and side flexion were normal and pain free. Flexion was relatively reduced (fingers to mid-tibia) and some spinal pain was reported at the end of range. Rising from flexion involved bracing of the lumbar spine and movement generation from the thoracic region and hips. Neurodynamic tests were unremarkable. Spinal palpation was pain free. The thoracic spine moved well and gave no pain responses. The hips moved well and quadrant testing was normal. Distal joint screening revealed no evidence of inflammation or synovitis.

### Impression & Treatment

Atypical movement patterns were evident but no focal neural concerns on testing in clinic, pointing to either central cord or nerve root pathology, were detected. The initial plan was to monitor and continue exercise-based physiotherapy input especially in light of recent gains from exercise.

Physiotherapy focused on functional movement quality with a trunk and lower limb strengthening programme. No formal myotome weakness was detected but the functional challenge when standing and the effortful nature of bridging was enough to warrant a programme to strengthen these tasks. Bridging was the main focus of the physiotherapy programme that involved review and progression of an exercise programme. Sit to stand exercises from a raised position were also encouraged. Reassurance was provided regarding the negative examination findings in clinic as evident fear and anxiety had been displayed in clinic and on the questionnaire responses. A review with the initial Extended Scope Practitioner (ESP) was advised if no further gains were made or any regressions materialised.

Some subjective gains were reported but objectively there were no gains after four sessions of treatment over a 2 month period. The ESP reviewed again. Isolated trunk and limb strength was re-assessed using myotome testing as well as isometric hip strength testing into abduction and extension. This again proved negative. Bridging remained effortful but the sit to stand movement was the main evident limitation.

An MRI of the entire spine was ordered by the ESP to assess the cord/neural health. If this test proved negative the next suggestion was to explore a neurology referral. The MRI hoped to differentiate or rule out the following possible origins to the bilateral lower limb weakness: spinal cord pathology such as compression from intervertebral discs, inflammation or intrinsic tumour, or more rarely a parasaggital brain lesion. The lack of upper motor neuron signs suggested a lower probability of any brain lesions though. Anterior horn cell disease could be ruled out and was low on the hypothesis list as the patient was young and did not have any fasciculations. Spinal canal stenosis and cauda equina change could also be examined but there was no bladder/sphincter symptoms or saddle changes so again the level of concern was low. A primary muscle disorder was another possible origin to be considered.

Blood tests ordered by the general practitioner had shown no significant change to inflammatory markers, urea, electrolytes or liver function. Repeat tests were scheduled by the general practitioner but did not include creatine kinase.

## Results

**Fig. 2:**
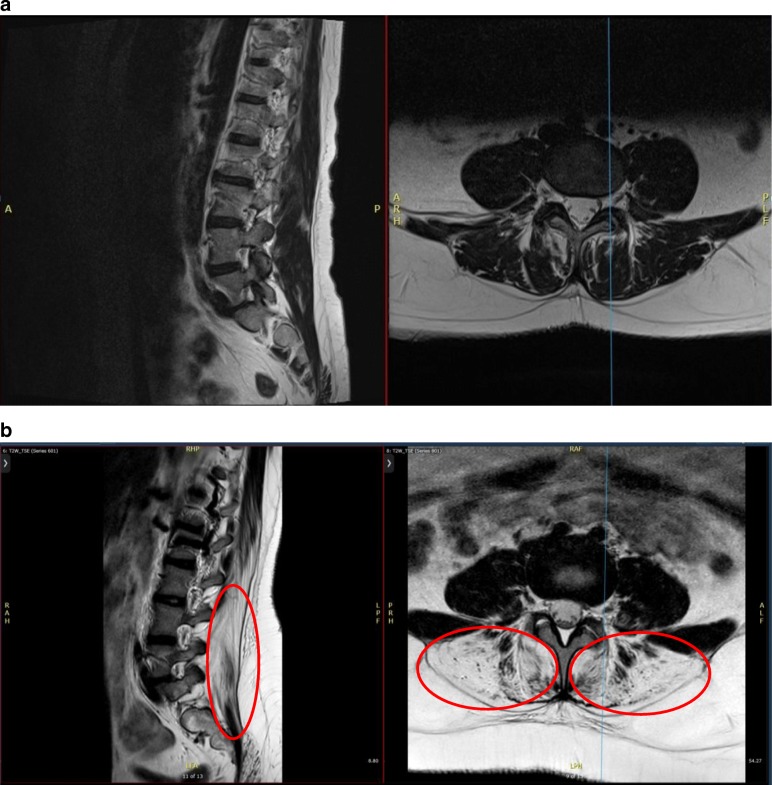
**a** Normal MRI. **b** Case report MRI images, sagittal and axial T2-weighted slices, from the lumbar spine demonstrate paraspinal muscle atrophy with fatty tissue replacing muscle, evident as high T2 signal, in the patient (circled)

The spinal MRI demonstrated severe selective atrophy and fatty replacement of the posterior paraspinal muscles within the lumbar spine, predominantly affecting the multifidus and erector spinae, extending from T12 to L5. This can be clearly seen in Fig. 2.

Serum creatine kinase was ordered following the MRI and was significantly elevated at 3398 IU/L (normal range 25–200).

At the neurology appointment 2 months after the last ESP review, proximal weakness of the lower limbs was evident. Electromyography of left vastus medialis demonstrated changes consistent with myopathy. A right quadriceps muscle biopsy showed dystrophic features. A clinical diagnosis of LGMD was made. The changes on muscle biopsy suggested a form of dystroglycanopathy. FKRP gene testing was negative. Genetic testing is ongoing for additional rare mutations that can cause this picture. Not all mutations have yet been identified.

## Discussion

### Limb Girdle Muscular Dystrophies (LGMDs)

There are many forms of muscular dystrophies. The LGMD category was initially defined in the 1950s to help classify cases that did not fit within the established diagnostic labels of the time, such as Duchenne, Becker and facioscapulohumeral dystrophies [5]. As a result, the range of pathophysiological states that present within the umbrella term of LGMD is wide-ranging [6]. Modern muscle biopsy histochemistry and advances in genetic testing have enabled many different forms of LGMD to be defined more precisely in recent years [7]. The goal of accurate genetic diagnosis is to guide genetic counselling and also to direct screening for respiratory and cardiac complications appropriately, which are features in some forms of LGMD and not others, and has been reported to improve quality and length of life [8].

The clinical hallmark of LGMD is muscular dystrophy with proximal weakness but no facial, distal limb or extraocular muscle involvement (at least in the early stages) [9]. Several LGMD symptoms may generate a referral to the physiotherapist for MSK related issues in undiagnosed individuals. These include weakness, decreased muscle bulk, scapular winging, fatigue, rippling of muscles, altered coordination, and hypertrophy of the calf muscles with pain in the calves [7, 8, 10–12]. Based on an extensive review of the literature, this is the first report of a case in which spinal pain and trunk weakness were the significant elements of the presentation to physiotherapy services that led to a subsequent diagnosis of LGMD.

Trunk muscle atrophy is reported in a study examining atrophy patterns in LGMD but was not assessed in all patients and the significance of the spinal atrophy was not elaborated upon [12]. Moderate to severe atrophy of the paraspinal muscles in the lumbar and thoracic spine was observed in 5 patients, but only 7 of the 25 LGMD subjects underwent spinal imaging. It would appear reasonable to predict that spinal pain might result from paraspinal muscular atrophy but individuals with severe scoliosis, yet no spinal pain, demonstrate that such biomechanical rationales do not always hold true [13].

On reflection, it was considered whether a creatine kinase measurement would have aided earlier diagnosis in this case. Creatine kinase is an enzyme present in skeletal muscle that can increase in blood following muscle damage. Elevated levels can be associated with muscular dystrophies but can be normal in some LGMD cases [7]. The blood test is not specific to LGMD. It is worth considering creatine kinase testing in patients with clinical pictures that are atypical of traditional MSK origins and especially if there is proximal weakness.

Subsequent diagnosis led to appropriate neurology based physiotherapy and occupational therapy input. The patient’s career routes could be considered. Specialist fertility services could be utilised if a genetic mutation is identified, to prevent LGMD being inherited by any potential offspring. The psychological implications of such a diagnosis should not be underestimated and are starkly highlighted in a paper examining whether an individual in Switzerland suffering from LGMD had the adequate muscular output required to take their own life or whether others had directly helped them [14]. Monitoring for cardiac and respiratory complications due to the muscular changes in the myocardium and respiratory muscles [15, 16] is an important aspect of management. A family history of cardiac or respiratory disorders can increase clinical suspicion of LGMD.

It must be stressed that many other neuromuscular conditions can present in a similar manner to LGMD, such as other types of muscular dystrophies, sporadic myopathies including treatable causes such as inflammatory myopathy, and mitochondrial disease [11]. The differentiation diagnosis of these particular dystrophies is beyond the scope of MSK physiotherapists and requires timely referral to a neurologist.

### Reflections

The integration of biological, psychological and social elements of an individual’s presentation has long been advised in the form of the biopsychosocial model instead of reductionist methods so that a holistic approach can be explored [17]. This case demonstrates such a holistic approach.

Examining such atrophy cases can pose a challenge for clinicians. Myotome testing has been shown to have inherent sensitivity limitations as demonstrated by levels as low as 0.22–0.4 in the cases of nerve root compression [5, 18]. Also, the traditional myotome assessment does not include testing of the gluteal muscles routinely, so assessment of bridging ability was also included as this correlated with the difficulty rising from sitting. In retrospect the use of more formal tools such as a handheld dynamometer may have enabled more measurable testing of hip extension at that point in time. Even with such a tool, a difference of 15% could still have escaped detection and given a falsely negative result [19].

For his patient, the atypical weakness, pain location, lack of response to conservative input as well as her young age category are defined red flags [20, 21]. Investigation is therefore justified in this context, following established guidelines [22, 23]. Whilst the MRI facilitated timely onward referral, diagnosis and management, this may not have been the initial test chosen if first evaluated in a different setting, such as a neurology clinic. As discussed above, the presence of atrophy on imaging is not the focus of investigation in LGMD and is not evident in everyone with the diagnosis. If imaging had proved negative, the opinion of a Neurologist would still have been explored. This is also true of the blood tests and formal strength testing as they are not sensitive enough to rely upon solely. The key consideration in this case report was the functional difficulty.

## Conclusion

The presented case report demonstrates appropriate use of the biopsychosocial model, judicious and timely investigation and early referral to enable the diagnosis of LGMD. This resulted in the diagnosis of a rare condition and subsequent appropriate management. Physiotherapy input could then be focused upon supportive methods to maximise independence, using assistive devices and enabling longevity of mobility. Consideration should be given to creatine kinase testing if a primary muscle disorder is suspected but is not always abnormal. Primary muscle disorders should be considered by MSK therapists if patients exhibit atypical features and non-specific musculoskeletal pain, for example, weakness, wasting, atypical gait patterns or progressive deterioration in symptoms. In such cases, there should be a low threshold for specialist referral.

### The patient’s clinical progress

Two years following diagnosis of LGMD, the patient now requires kitchen aids to prepare meals. Mobilisation is aided by 2 elbow crutches. A wheelchair is needed for longer distances. Two falls have been reported on the stairs due to progression of weakness in the lower limbs. A raised toilet seat, perching stool and riser chair are used. Psychological support has been recommended. Regular attendance to the local gym has been encouraged and enjoyed. Testing for rare genetic mutations is ongoing through the national referral centre.

## Supplementary information

**Additional file 1.** STarT Back Actual Form (completed by referring GP)

**Additional file 2.** EQ5D Questionnaire responses at initial appointment

## Data Availability

Not applicable.
