# Supplementary material for: Limb girdle muscular dystrophy: a case report initially presenting to an outpatient musculoskeletal physiotherapy clinic with spinal pain and functional weakness
Source: Arch Physiother. 2019 Nov 14;9:13. doi: 10.1186/s40945-019-0066-3 (PMC6857131; doi:10.1186/s40945-019-0066-3)
Supplement: Supplementary file 1 — Additional file 1. STarT Back Actual Form (completed by referring GP) [file 40945_2019_66_MOESM1_ESM.docx]

Additional file 1: STarT Back Actual Form (completed by referring GP)
